# Association Between Arterial Stiffness and Heart Failure With Preserved Ejection Fraction

**DOI:** 10.3389/fcvm.2021.707162

**Published:** 2021-08-11

**Authors:** Chen Chi, Yifan Liu, Yawei Xu, Dachun Xu

**Affiliations:** Department of Cardiology, Shanghai Tenth People's Hospital, Tongji University School of Medicine, Shanghai, China

**Keywords:** arterial stiffening, heart failure, HFpEF, aging, HFmrEF—heart failure with mid-range ejection fraction

## Abstract

Cardiovascular diseases are the leading cause of mortality in the world. Heart failure with preserved ejection fraction (HFpEF) accounts for about half of all heart failure. Unfortunately, the mechanisms of HFpEF are still unclear, leading to little progress of effective treatment of HFpEF. Arterial stiffness is the decrement of arterial compliance. The media of large arteries degenerate in both physiological and pathological conditions. Many studies have proven that arterial stiffness is an independent risk factor for cardiovascular disorders including diastolic dysfunction. In this perspective, we discussed if arterial stiffness is related to HFpEF, and how does arterial stiffness contribute to HFpEF. Finally, we briefly summarized current treatment strategies on arterial stiffness and HFpEF. Though some new drugs were developed, the safety and effectiveness were not adequately assessed. New pharmacologic treatment for arterial stiffness and HFpEF are urgently needed.

## Introduction

Cardiovascular diseases are the leading cause of mortality in the world. Heart failure (HF), as the consequence of so many factors that damage the heart, is a progressive and serious condition with high rate of mortality (1). Heart failure with preserved ejection fraction (HFpEF) accounts for about half of heart failure patients. The prevalence of HFpEF significantly associated with age, and age-related diseases like hypertension and coronary artery disease (2). In people over or equal to 60 years old, ~5% of them are with HFpEF (3). Moreover, the estimated 5-year survival rate is only about 50% (4). HFpEF has been a global health problem, and there is an urgent need for physicians to understand the pathology and treatment of HFpEF.

Arterial stiffness is the decrement of arterial compliance. A lot of parameters were used in clinical practice to assess arterial stiffness (5), for example, pulse pressure (PP) and its amplification (PPA), pulse wave velocity (PWV), augmentation index (AIx), etc. For large arteries like aorta, the media of these arteries degenerate in both physiological conditions like aging and pathological conditions like chronic inflammation, oxidative stress, hypertension, diabetes, etc., resulting in the stiffness of these arteries (6). Numerous studies have proven that arterial stiffness is an independent risk factor for cardiovascular diseases, events, and mortality. Moreover, the relationship between arterial stiffness and left ventricular (LV) diastolic function is verified by dozens of observational studies (7).

Because arterial stiffness is closely related to LV diastolic function, physicians are paying great attention to the contribution of arterial stiffness to HFpEF. In this perspective, we summarized current understanding of the relationship between arterial stiffness and HFpEF, the mechanism of arterial stiffness contributing to HFpEF, and the treatment and potential future directions of HFpEF focusing on arterial stiffness.

## Does Arterial Stiffness Relate to HFpEF?

Many conditions were reported to be associated with HFpEF, for example, aging, hypertension, obesity, diabetes. Diastolic dysfunction is one of the most important contributor to HFpEF (8), and arterial stiffness is a well-established factor that accelerates the development of diastolic dysfunction. A meta-analysis which included 27 studies showed that, parameters of arterial stiffness especially brachial-ankle PWV, were significantly associated with diastolic dysfunction indicators recorded by echocardiography (9). Pulse pressure is another indicator for arterial stiffness. Data from Mayo Clinic showed that, both central and brachial pulse pressure were significantly associated with diastolic dysfunction assessed by echocardiography (10). Apart from these indirect links, many studies also showed direct or independent associations between arterial stiffness and HFpEF. Compared with hypertensive controls, HFpEF patients were with reduced total arterial compliance (11). A study included 60 HFpEF patients and 51 non-HFpEF controls showed that, compared with patients without HFpEF, brachial-ankle PWV was higher in HFpEF patients. Besides, arterial stiffness was strongly associated with cardiovascular events during the median follow-up period of 54 months in this study (12). Apart from hard endpoints, a study conducted in Japan showed that cardio-ankle vascular index, another arterial stiffness parameter, was independently and significantly associated with hospitalization of HFpEF patients after adjustment for hypertension, diabetes, and renal function (13). It seemed that the difference of arterial stiffness in patients with/without HFpEF was more likely to be observed during exercise (14, 15).

Although many studies reported the associations among arterial stiffness, diastolic dysfunction, and HFpEF, till now there is very few evidence to prove that arterial stiffness is a key factor which drives the development of HFpEF. A study conducted by Wan et al. (16) showed that, in 488 hypertensive patients with HFpEF, two arterial stiffness parameters, arterial pressure volume index (API) and arterial velocity pulse index (AVI), were both significantly associated with the onset of HFpEF. Another case control study which included 77 matched pairs demonstrated that, participants with decreased aortic distensibility were more easily to develop HFpEF with asymptomatic diastolic dysfunction (17). However, negative association between arterial stiffness and HFpEF was also reported. In the Health ABC study, the authors divided 2,290 elderly participants into three groups based on the tertiles of PWV measured at baseline. This study demonstrated that, after adjustment for conventional cardiovascular risk factors, compared to participants with low PWV (tertile-1), participants with high PWV (tertile-3) were not significantly associated the high risk of HFpEF with the mean follow-up time of 11.2 years (18). More large prospective studies are warranted to further investigate the relationship between arterial stiffness and HFpEF.

In conclusion, though there is a little controversy, arterial stiffness is more likely to be regarded as a harmful factor for LV diastolic function and HFpEF. Especially for the arterial stiffness induced by metabolic disorders, it may play a major role in the development of HFpEF.

## How Does Arterial Stiffness Contribute to HFpEF?

As we described previously, many studies have demonstrated that the magnitude of arterial stiffness in HFpEF is significantly increased compared to those without HFpEF. This accelerated arterial stiffness leads to the increment of arterial pulse pressure and LV afterload (19, 20). This increment was further amplified in specific conditions like hypertension, diabetes, and exercise (15). Several mechanisms were related to the contribution of arterial stiffness to HFpEF, including: (1) the vascular effect, (2) the ventricular–vascular interaction effect, (3) the effect on arterial hemodynamics, and (4) the linkage between renal function, arterial stiffness, and HFpEF. Cellular and molecular mechanisms are focusing on endothelial cells currently.

The vascular effect of arterial stiffness on HFpEF is similar to other organ damage induced by arterial stiffness. The former leader of the vascular group of Framingham study, Professor Mitchell, proposed a theory of “arteriosclerosis-related organ damage” (21). In this theory, pulse pressure becomes high owning to arterial stiffness. Since stiff arteries cannot adequately absorb the pulse energy from blood pressure, target organs suffered more redundant energy from pulse pressure, and subsequently got damaged from this energy. Small arteries in heart were also damaged in this way. Besides, the diastole is the most important time duration for coronary flow, and the leading force for coronary perfusion is diastolic pressure. When arterial stiffness occurs, diastolic pressure become lower than the normal (22). This low diastolic pressure is not enough for coronary perfusion. Thus, systolic pressure may become the major force for perfusion, which makes the heart more sensitive to systolic disorders (23).

The change of arterial hemodynamics plays a key role in HFpEF. Arterial stiffness is a determinant of pulsatile afterload, which is one of the two major components of arterial load. Arterial wave reflections increases with arterial stiffness, leading to the increment of mid-to-late systolic load, and subsequent left ventricular abnormalities including concentric remodeling, myocardial fibrosis, contractile dysfunction, and ejection duration reduction. These changes further contribute to the increment of mid-to-late systolic load, resulting in a vicious circle (24). There might be a critical linkage between renal dysfunction, arterial stiffness, and HFpEF. Renal dysfunction results in the calcification of arterial wall and the stiffness of arteries. Tremendous studies have proved that renal dysfunction are closely related to arterial stiffness, and it has good value in prediction of mortality (25). However, the direct evidence among HFpEF and renal dysfunction needs obtaining in the future.

The ventricular–vascular interaction effect is more complicated. In HFpEF, not only arteries but also left ventricle become stiff. The end-systolic elastance (Ees, defined as the slope and intercept of end-systolic pressure and the difference of end-systolic volume with initial volume) and arterial elastance (Ea, defined as the slope of end-systolic pressure and stroke volume) are the indicators for left ventricle systolic stiffness and arterial stiffness, respectively. In resting situations, though both Ees and Ea elevated in HFpEF, stroke volume and pulse energy are close to physiological conditions. However, since the slopes of Ees and Ea are sharp, a small change in blood pressure may lead to a dramatic change in Ees and Ea, leading to the mismatch between left ventricle and arteries (26). This ventricular-vascular interaction effect may explain why exercise amplified the clinical measurements of arterial stiffness in patients with/without HFpEF (27).

Endothelial function plays an important role in arterial stiffness and HFpEF. The relationship between endothelial dysfunction and HFpEF is well-established by a lot of studies (28). Endothelial function not only tightly associates with HFpEF, but also strongly predicts events in HFpEF patients (29). This is because endothelial cells: (1) participate in anti-oxidative and anti-inflammatory activities in arteries; (2) interact with extracellular matrix (Elastic and collagen fibers) to regulate vascular elasticity; (3) directly affect vascular tone by synthesizing and releasing nitric oxide (30). The Sirtuin family, especially sirt1 and sirt3, play key roles in the regulation of endothelial function in HFpEF (31). Functions of other cells like vascular smooth muscle cells and macrophages may be involved in arterial stiffness and HFpEF, but more evidences are needed (32).

## Does Treatment for Arterial Stiffness Benefit in HFpEF?

**Figure 1:**
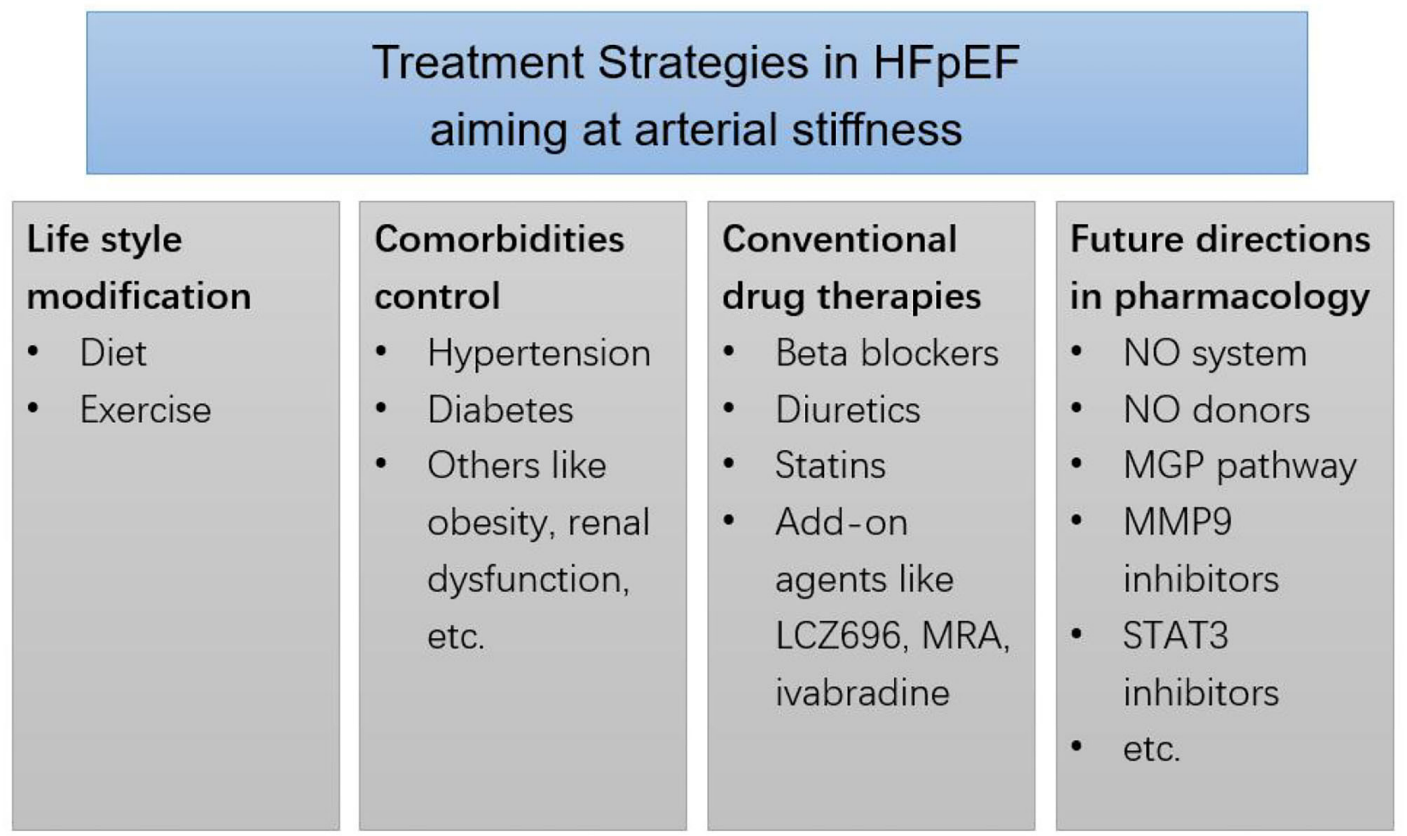
Treatment strategies in HFpEF aiming at arterial stiffness. MRA, mineralocorticoid receptor antagonists. MGP, matrix gla-protein. MMP9, matrix metalloproteinases 9. STAT3, transcription factor signals transducer and activator of transcription 3.

Unlikely to the treatment of heart failure with reduced EF, there is no dramatic progress in the treatment of HFpEF, and the survival rate of HFpEF patients is not significantly improved (33). Thus, looking for the new approach for the management of HFpEF is necessary. Because of the contribution of arterial stiffness to HFpEF, therapies aiming at arterial stiffness may be helpful to HFpEF. Here, we briefly discussed the treatment of arterial stiffness and HFpEF from five aspects, that is, lifestyle management, comorbidities control, conventional anti-hypertensive treatment, recent advances, and future directions (Figure 1).

Lifestyle management is fundamental to HFpEF patients. Proper diet and exercise are of critical importance. The continuities between good diet and cardiovascular health are obvious. For example, the sodium-restricted diet was able to improve ventricular-arterial coupling together with the arterial elastance of hypertensive HFpEF patients (34). Exercise is helpful to treat HFpEF and arterial stiffness. The beneficial effects of exercise may be owing to the improvement of oxygen utilization and exercise capacity (35, 36). Diet and exercise can also modulate risk factors like obesity, hypertension, diabetes apart from the direct beneficial effects to the heart and arteries.

The control of comorbidities is also important in arterial stiffness and HFpEF. We take hypertension and diabetes as examples. Hypertension is a major risk factor of HFpEF. According to the recommendations of guidelines, blood pressure control of HFpEF hypertensive patients should be strict and even aggressive (33). Long-term blood pressure lowing therapy is able to reduce arterial stiffness and cardiovascular events (37). Given the fact that diabetes is related to both arterial stiffness and HFpEF, the effect of diabetic control should be considered. Currently, several kinds of anti-diabetic drugs can affect arterial stiffness, for example, glucagon-like peptide-1 receptor agonist (GLP-1 RA), and sodium-glucose cotransporter-2 inhibitors (SGLT-2i). GLP-1 RA and SGLT-2i have been proven to protect against cardiovascular events (38).

Conventional anti-hypertensive agents include renin-angiotensin-aldosterone system (RAAS) blockades, beta-blockers, calcium channel blockers, and diuretics. No matter which mechanism is, arterial stiffness parameters like PWV could be reduced by most kinds of anti-hypertensive drugs. However, these drugs have their own features. RAS blockades are reported to protect from the change of vascular structure and subsequent arterial stiffness, other drugs may reduce PWV because of their influence on hemodynamics (39). But effects of RAS blockades on HFpEF is controversial. Neither angiotensin converting enzyme inhibitor (ACEI) perindopril (40) nor angiotensin receptor blocker (ARB) irbesartan (41) improved mortality in HFpEF patients. Thus, the use of ACEI or ARB for direct treatment of HFpEF is not supported by evidence. A recent meta-analysis which included 10 trials investigating beta-blockers showed that, beta-blockers might reduce cardiovascular mortality in HFpEF patients, but the evidence certainty was low (42). Till now, there is no large prospective trials focusing on calcium channel blockers in HFpEF. One ongoing clinical trial that tests the effect of nifedipine on HFpEF is found in clinicaltrials.gov (NCT01157481). Diuretics might be necessary in HFpEF patients. Despite their significant effects on symptoms in HFpEF patients, results from the ALLHAT trial suggested that, compared to amlodipine, lisinopril, or doxazosin, chlorthalidone significantly reduced the occurrence of new-onset HFpEF (43). Even these drugs did not show great superiority in HFpEF treatment, current studies did not find any disadvantages of anti-hypertensive agents for hypertensive HFpEF patients at least. And as we mentioned before, blood pressure lowing therapy has a lot of beneficial effects. The optimal and individualized anti-hypertensive strategy should be applied to accomplish effective blood pressure control. Besides, it should be pointed out that, though some drugs have effects on both arterial stiffness and HFpEF, it is unclear whether these drugs affect HFpEF through arterial stiffness.

Some advances were made in recent years. Though ACEI/ARB did not show much protective effect on HFpEF, the inhibition of RAAS is still an important approach to the management of HFpEF patients. Sacubitril/Valsartan (LCZ696) is a new superstar in the management of heart failure and hypertension. Two recently-released independent studies showed that sacubitril/valsartan inhibits the progress of diastolic dysfunction and arterial stiffness to HFpEF in rat models (44, 45). However, clinical trials are needed to verify the effects of sacubitril/valsartan on HFpEF patients. Mineralocorticoid receptor antagonists (MRA) are important in RAAS inhibition. Current evidence from the TOPCAT trial suggested that, spironolactone could be used as an add-on therapy rather than initial therapy for HFpEF patients, especially for those with resistant hypertension (46, 47). Heart rate control is another important issue for heart failure patients, and heart rate is closely associated with PWV. Apart from beta blockers, If-channel inhibitor ivabradine can reduce heart rate and improve diastolic function. However, Komajda et al. (48) found that ivabradine had no effect on E/e′ and NT-proBNP level in HFpEF patients compared to placebo. Statins are fundamental for patients with atherosclerosis, a meta-analysis showed that statin therapy may improve mortality rate of HFpEF patients (49).

As for the new treatment strategy for arterial stiffness, Tsai et al. (50) summarized nine directions for future research. A few of them are with limited data about the effect on heart failure currently, these are: (1) NO system (51), (2) NO donors (52), (3) the Matrix Gla-Protein (MGP) pathway (53), (4) Matrix Metalloproteinases (MMP) 9 Inhibitors (54), and (5) transcription factor signals transducer and activator of transcription (Stat) 3 inhibitors (55). However, these strategies are lack of clinical data so that cannot be used extensively [please refer the review by Athyros et al. (56) for the detail].

## Conclusion

HFpEF accounts for about half of the total heart failure. However, the understand of HFpEF is poor. Arterial stiffness is a well-established cardiovascular risk factor, and it is able to accelerate the pathogenesis and development of diastolic dysfunction. Mechanisms of the contribution of arterial stiffness to HFpEF are owing to the vascular effect, the ventricular-vascular interaction effect, and the linkage of renal function. Endothelial cells play key roles in this process. Unfortunately, current therapy on HFpEF did not significantly improve the mortality in HFpEF patients. Therapies aiming at arterial stiffness may become a new strategy for the improvement of HFpEF treatment in the future.

## Data Availability Statement

The original contributions presented in the study are included in the article/supplementary material, further inquiries can be directed to the corresponding author/s.

## Author Contributions

DX: conception and design. CC and YL: manuscript writing. DX, CC, YL, and YX: final approval of manuscript. All authors contributed to the article and approved the submitted version.

## Conflict of Interest

The authors declare that the research was conducted in the absence of any commercial or financial relationships that could be construed as a potential conflict of interest.

## Publisher's Note

All claims expressed in this article are solely those of the authors and do not necessarily represent those of their affiliated organizations, or those of the publisher, the editors and the reviewers. Any product that may be evaluated in this article, or claim that may be made by its manufacturer, is not guaranteed or endorsed by the publisher.
